# Association between *MPHOSPH6* gene polymorphisms and IgA nephropathy risk in a Chinese Han population

**DOI:** 10.18632/oncotarget.19758

**Published:** 2017-08-01

**Authors:** Xiaohong Yang, Yin Zhang, Wenning Li, Yan Su, Dan Niu, Yanni Wang, Haiyang Huang, Hui Han, Daofa Zhang, Maowei Xie, Huiluan Su, Wentan Xu, Jiali Wei

**Affiliations:** ^1^ Department of Nephrology, Hainan General Hospital, Haikou Hainan 570311, China; ^2^ Department of Nephrology, The First Affiliated Hospital of Xi'an Jiaotong University, Xi'an 710061, China; ^3^ Central Laboratory, Hainan General Hospital, Haikou Hainan 570311, China

**Keywords:** IgA nephropathy (IgAN), MPHOSPH6, single nucleotide polymorphisms (SNPs), association study

## Abstract

Multiple genetic and environmental factors together contribute to the risk of IgA nephropathy (IgAN). *MPHOSPH6* play an important role in the recruitment of the exosome to the pre-rRNA. However, to date, little information is found about the association between *MPHOSPH6* polymorphisms and the IgAN risk. In this case-control study, we genotyped five single nucleotide polymorphisms (SNPs) in *MPHOSPH6* gene in 416 IgAN cases and 495 controls using Sequenom Mass-ARRAY technology and evaluated their association with IgAN using the χ2 and genetic model analysis. In the allelic model analysis, we determined rs1056654 was associated with a 0.774-fold decrease in the risk of IgAN (95%CI= 0.630-0.952; *p* = 0.015). In the genetic model analysis, we found that the “C/C” genotype of rs1056675 was associated with an increased risk of IgAN based on the codominant model (OR =1.48; 95% CI=1.03-2.13; *p*=0.033) and recessive model (OR =1.52; 95% CI=1.11-2.09; *p*=0.0095). The “G/A-A/A” genotype of rs1056654 was associated with a decreased risk of IgAN based on the dominant model (OR =0.75; 95% CI=0.58-0.98; *p*=0.032) and log-additvie model (OR =0.78; 95% CI=0.64-0.96; *p*=0.0188). Our data suggested that gene polymorphisms in the *MPHOSPH6* may exert influences IgAN susceptibility in a Chinese Han population.

## INTRODUCTION

IgA nephropathy (IgAN) is the most common type of primary glomerulonephritis [1], and more than 40% of IgAN patients will eventually worsen to end stage renal disease [2]. However, the prevalence of IgAN is significantly different among ethnicities. It is most common in individuals of Asian ancestry, with a very high prevalence of 3.7% [3], but is rare among Africans [4] and is of intermediate prevalence among Europeans (up to 1.3%) [5]. It has been widely accepted that multiple genetic and environmental factors together contribute to the risk of IgAN.

In the past decades, several genome-wide association studies (GWAS) have linked a plenty variety of genetic abnormality in the onset and development of IgAN. Among them, complement factor H related 1 and 3 (*CFHR1*, *CFHR3*) [6], tumor necrosis factor (*TNFSF13*) [7] and a-defensin (*DEFA*), major histocompatibility complex (*MHC*) [8] genes polymorphisms were the most common susceptibility genes with the risk of IgAN. Recently, microRNAs such as miR-148b [9], miR-n-9 [10] were associated with the development of IgAN. These results remind us that study the polymorphisms of genes involving in formation of RNA may provide us a better understanding of IgAN.

It has been demonstrated that *MPHOSPH6* play an important role in the recruitment of the exosome to the pre-rRNA [11]. *MPHOSPH6* also might be involved in regulating shrimp cell cycle and ovary development [12]. However, to date, little information is found about the association between *MPHOSPH6* polymorphisms and the risk of IgAN. In the present study, we selected five single nucleotide polymorphisms (SNPs) of *MPHOSPH6* to investigate the association between *MPHOSPH6* polymorphisms and the risk of IgAN in a Chinese Han population.

## RESULTS

A total of 416 IgAN cases (271 men and 145 women; mean age, 33.35 ±12.13 years) and 495 controls (180 men and 315 women; mean age, 54.48 ±9.44 years) were included in the study. The basic characteristics of the cases and controls are shown in Table 1.

**Table 1:** Characteristics of cases and controls included in this study

Variables	Case (N=416)	Control (N=495)	*p*-value
Sex, No.(%)			< 0.001^a^
Male	271	180	
Female	145	315	
Mean age ±SD	33.35 ±12.13	54.48 ±9.44	< 0.001^b^

^a^ The *p* value was calculated from Pearson's chi-square tests.

^b^ The *p* value was calculated by Welch's t tests.

SD, standard deviation.

The MAFs of the analyzed SNPs in the case and control groups are shown in Table 2. All SNPs were in Hardy-Weinberg equilibrium (HWE) in the controls (*p* > 0.05) with the exception of rs1056629, which was excluded from subsequent analyses. The MAFs of the SNPs in the control group were similar to those reported for the HapMap Asian population. Using chi-square tests, we determined that rs1056654 was associated with a 0.774-fold decrease in the risk of IgAN (95% confidence interval [CI] = 0.630-0.952; *p* = 0.015).

**Table 2:** Allele frequencies in cases and controls and odds ratio estimates for IgA nephropathy

SNP ID	Band	Position	Gene	Alleles A^a^/B	MAF		HWE *p*-value	ORs(95%CI)	*p*-value
					Case	Control			
rs1056675	16q23.3	82181934	*MPHOSPH6*	C/T	0.476	0.430	1	1.203(0.999-1.449)	0.051
rs1056654	16q23.3	82182011	*MPHOSPH6*	A/G	0.252	0.304	0.243	0.774(0.630-0.952)	0.015*
rs1056629	16q23.3	82182104	*MPHOSPH6*	C/T	0.252	0.251	0.017^#^	1.010(0.817-1.249)	0.926
rs3751862	16q23.3	82182229	*MPHOSPH6*	C/A	0.041	0.050	1	0.817(0.521-1.277)	0.374
rs2967361	16q23.3	82203503	*MPHOSPH6*	T/G	0.234	0.223	0.069	1.065(0.855-1.326)	0.573

MAF, minor allelic frequency; HWE, Hardy-Weinberg Equilibrium; ORs, odds ratios; CI: confidence interval.

^a^ Minor allele; ^#^ HWE *p*-value ≤ 0.05 was excluded; *p value ≤ 0.05 indicates statistical significance.

The genotype frequencies of the *MPHOSPH6* polymorphisms are shown in Table 3. Compared with the TT genotype, the CC frequency of rs1056675 polymorphism among cases was significantly different from controls (CC versus TT: OR =1.48, 95% CI=1.03-2.13, *p*=0.035), which suggested that the rs1056675 polymorphism had an increased effect on the risk of IgAN. Additionally, we observed that rs1056654 polymorphism have a protective role of IgAN risk (AA versus GG: OR =0.61, 95% CI=0.38-0.98, *p*=0.045).

**Table 3:** Genotypes frequencies of the SNPs and their associations with risk of IgA nephropathy

SNP ID	Genotype	Genotype Frequencies		OR(95%CI)	*p*^a^
		Case	Control		
rs1056675	TT	126(30.4%)	160(32.5%)	1.00	
	CT	182(44.0%)	242(49.0%)	0.96(0.71-1.29)	0.765
	CC	106(25.6%)	91(18.5%)	1.48(1.03-2.13)	0.035*
rs1056654	GG	236(56.7%)	245(49.6%)	1.00	
	AG	150(36.1%)	198(40.1%)	0.79(0.60-1.04)	0.090
	AA	30(7.2%)	51(10.3%)	0.61(0.38-0.98)	0.045*
rs3751862	AA	382(91.8%)	446(90.3%)	1.00	
	CA	34(8.2%)	47(9.5%)	0.85(0.53-1.34)	0.474
	CC	0(0%)	1(0.2%)	/	/
rs2967361	GG	244(58.7%)	306(61.8%)	1.00	
	TG	149(35.8%)	157(31.8%)	1.19(0.90-1.58)	0.223
	TT	23(5.5%)	32(6.4%)	0.90(0.51-1.58	0.717

SNP: Single nucleotide polymorphism; OR: odds ratio; 95%CI: 95% confidence interval.

^a^ *p* values were calculated by unconditional logistic regression analysis with adjustments for age and gender.

*p≤0.05 indicates statistical significance.

Furthermore, we assumed that the minor allele of each SNP as a risk factor compared with the wild-type allele. Four models (codominant, dominant, recessive, and log-additive) were applied to analyze the associations between the SNPs and risk of IgAN using an unconditional logistic regression analysis with adjustments for age and sex. We found that the “C/C” genotype of rs1056675 was associated with an increased risk of IgAN based on the codominant model (OR =1.48; 95% CI=1.03-2.13; *p*=0.033) and recessive model (OR =1.52; 95% CI=1.11-2.09; *p*=0.0095). The “G/A-A/A” genotype of rs1056654 was associated with a decreased risk of IgAN based on the dominant model (OR =0.75; 95% CI=0.58-0.98; *p*=0.032) and log-additvie model (OR =0.78; 95% CI=0.64-0.96; *p*=0.0188) (Table 4).

**Table 4:** Association between significant SNPs and risk of IgA nephropathy in multiple inheritance models

SNPs	Model	Genotype	Case	Control	OR (95% CI)	*p*-value	AIC	BIC
rs1056675	Codominant	T/T	126 (30.4%)	160 (32.5%)	1			
		T/C	182 (44.0%)	242 (49.1%)	0.96 (0.71-1.29)	0.033*	1249.7	1264.1
		C/C	106 (25.6%)	91 (18.5%)	**1.48 (1.03-2.13)**			
	Dominant	T/T	126 (30.4%)	160 (32.5%)	1	0.51	1254.1	1263.7
		T/C-C/C	288 (69.6%)	333 (67.5%)	1.10 (0.83-1.46)			
	Recessive	T/T-T/C	308 (74.4%)	402 (81.5%)	1	0.0095*	1247.7	1257.4
		C/C	106 (25.6%)	91 (18.5%)	**1.52 (1.11-2.09)**			
	Log-additive	—	—	—	1.19 (0.99-1.43)	0.057	1250.9	1260.5
rs1056654	Codominant	G/G	236 (56.7%)	245 (49.6%)	1			
		G/A	150 (36.1%)	198 (40.1%)	0.79 (0.60-1.04)	0.06	1255.2	1269.7
		A/A	30 (7.2%)	51 (10.3%)	0.61 (0.38-0.99)			
	Dominant	G/G	236 (56.7%)	245 (49.6%)	1	0.032*	1254.2	1263.8
		G/A-A/A	180 (43.3%)	249 (50.4%)	**0.75 (0.58-0.98)**			
	Recessive	G/G-G/A	386 (92.8%)	443 (89.7%)	1	0.098	1256.1	1265.7
		A/A	30 (7.2%)	51 (10.3%)	0.68 (0.42-1.08)			
	Log-additive	—	—	—	**0.78 (0.64-0.96)**	0.0188*	1253.2	1262.8

ORs, odds ratios; CI: confidence interval; AIC: Akaike's Information criterion; BIC: Bayesian Information criterion.

* *p* value ≤0.05 indicates statistical significance.

## DISCUSSION

In this study, we investigated the associations between five selected *MPHOSPH6* SNPs and risk of IgAN in the Chinese Han population of Shaanxi Province. We found that rs1056675 is associated with an increased risk of IgAN, while rs1056654 has a protective role for IgAN. Our results suggest that the polymorphisms of *MPHOSPH6* may play an important role in the risk of IgAN in the Chinese Han population.

*MPHOSPH6* is located on chromosome 16q23.3 and encodes the M-phase phosphoprotein 6 (MPP6) that is important for the maturation of 5.8S rRNA. MPP6 is also a RNA-binding protein, which preferentially binds to pyrimidine homopolymers [13]. In fact, little study is found about the association between *MPHOSPH6* and disease. Li et al has attempted to investigate the association between *MPHOSPH6* polymorphisms and risk of colorectal cancer, but they have no significant result [14]. In our study, we detected five SNPs of *MPHOSPH6*, and found that genetic polymorphisms of *MPHOSPH6* are associated with IgAN, which may shed a new light on the in-depth study for this gene.

Previous association studies have found many SNPs associated with IgAN risk in some populations; however, little of them were successfully replicated in other populations. Earlier GWAS studies revealed two common variants rs3115573 and rs3130315 in *MHC* that influence IgAN risk in a British population [15]. Chinese researchers also attempted to investigate SNPs associated with IgAN in the Chinese populations, they identified novel SNPs including rs3803800, rs2738048, rs660895, rs1794275 and rs2523946 reached a genome-wide significance [8]. It has been revealed that four SNPs including rs9275224, rs2856717, rs9275424 and rs9275596 were significantly associated with IgAN risk in a GWAS replication study in a combined Chinese, European and African-American population and followed by a meta-analysis in 85 world populations [16]. In the present study, we found that rs1056675 and rs1056654 were significantly associated with IgAN risk in a Chinese Han population. As far as we know, we are the first to report the association between *MPHOSPH6* polymorphisms and IgAN risk, so the results identified here should be confirmed in further studies.

Our study had several intrinsic limitations. Firstly, the subjects in this study were all Han Chinese who lived in Shaanxi Province. The results identified here need to be confirmed in other ethnicities. Secondly, our sample size was relatively small, so we didn't do further subgroup analyses based on age or gender. Thirdly, IgAN is a heterogeneous disease with many other risk factors. We could not explore the interactions between genetic polymorphisms and environmental factors in IgAN patients due to the limited data. Therefore, the relationship between *MPHOSPH6* polymorphisms and IgAN risk must be evaluated in future studies with bigger sample size and different populations.

## MATERIALS AND METHODS

### Subjects

We consecutively recruited 416 IgAN cases and 495 healthy controls from the First Affiliated Hospital of Xi'an Jiaotong University from March 2014 to December 2016. All subjects were of northern Han Chinese ancestry and were recruited among local inhabitants of Shanxi province from northern China. All the patients were diagnosed and histologically confirmed to suffer from IgAN according to the renal biopsy, and they had not received any systemic treatment before the time of examination. Besides, we excluded patients with any type of cancer, infection, secondary IgAN (Secondary IgAN is seen most commonly in patients with liver disease or mucosal inflammation, in particular affecting the gastrointestinal tract), other renal diseases or autoimmune diseases. We also excluded control subjects with any chronic disease, conditions involving vital organs (liver, heart, lung and brain), central nervous system-related disease, or aggressive metabolic and endocrinological disease. Peripheral blood was collected from both cases and controls for DNA extraction.

All of the participants provided written informed consent. The Human Research Committee for Approval of Research Involving Human Subjects, the First Affiliated Hospital of Xi'an Jiaotong University, approved the use of human blood samples in this study.

### SNP selection and genotyping

In this study, five SNPs in *MPHOSPH6* were selected from DbSNP database (http://www.hapmap.org/index.html.en) and SNP Consortium database (http://snp.cshl.org/) for analysis. The lower frequency alleles were coded as the minor allele. All of the SNPs had minor allele frequencies (MAFs) > 5% in the HapMap Chinese Han Beijing population. Genomic DNA was isolated from whole blood samples using the GoldMag-Mini Purification Kit (GoldMagCo. Ltd. Xi'an, China), and DNA concentrations were measured using the NanoDrop2000 (Thermo Scientific, Waltham, MA, USA). Sequenom Massarray Assay Design 3.0 softwarewas used to design a multiplexed SNP Mass EXTENDED assay [17–19]. Genotyping was performed on a Sequenom MassARRAY RS1000 platform using the manufacturer's protocol. The PCR primers for each SNP are shown in Table 5. Data management and analysis was performed using the Sequenom Typer 4.0 Software [20, 21].

**Table 5:** Primers used in this study

SNP	1st_PCR primer	2nd_PCR primer	UEP_SEQ
rs1056675	ACGTTGGATGAATACTTAAGGCTGGAGAGG	ACGTTGGATGGTCAAGCCAATTCGTACATAC	ggtgCGTACATACAATTTGGAATCAA
rs1056654	ACGTTGGATGGTATGTACGAATTGGCTTGAC	ACGTTGGATGCAGTCACTGACCTTGAATTG	ACCTTGAATTGACTTACATAAA
rs1056629	ACGTTGGATGTTTTTAGCCCCTGATCTAC	ACGTTGGATGGGTCAGTGACTGGAGAACTA	cGGAAGCAGCCCTGTAACAA
rs3751862	ACGTTGGATGTGGTGTCTCTATAGTTATT	ACGTTGGATGCATCTGTTTCAAAAACAGC	TGTTTCTAAAATGATAATCTCTTTACA
rs2967361	ACGTTGGATGTTACTGGGAACCAGCTTACG	ACGTTGGATGAGCTGTACCCTGACTGCTTC	tCCTGACTGCTTCTGTGTAC

UEP_SEQ: Unextended mini-sequencing primer.

### Statistical analysis

We used Microsoft Excel (Microsoft Corporation, Redmond, WA, USA) and the SPSS 18.0 statistical package (SPSS, Chicago, IL, USA) to perform statistical analyses. All *p* values presented in this study were two sided, and *p* = 0.05 was considered the cutoff for statistical significance. Differences in the characteristics of the case and control study populations were analyzed using chi-square tests for categorical variables and Welch's t tests for continuous variables. In all analyses, the lower frequency allele was considered to be the ‘risk’ allele. Control genotype frequencies for each SNP were tested for departure from HWE using Fisher's exact tests. Allele and genotype frequencies in the cases and controls were compared using chi-square tests [22]. Four models (codominant, dominant, recessive, and log-additive) were used to assess the association between each genotype and the risk of IgAN. The effects of the polymorphisms on the risk of IgAN were expressed as odds ratios (ORs) with 95% confidence interval (CIs), which were calculated using unconditional logistic regression analysis after adjusting for age and gender [23].

## CONCLUSIONS

The present study provided evidence that SNPs in the *MPHOSPH6* are associated with IgAN in a Chinese Han population. It is possible that these variants are IgAN risk factors and these data can provide a theoretical foundation for other researchers to further study the association between the *MPHOSPH6* gene and IgAN risk in the Chinese Han or other populations.
